# Subacute Osteomyelitis of the Pediatric Talus: A First Report of Brodie's Abscess from Morganella morganii

**DOI:** 10.1155/2019/7108047

**Published:** 2019-03-11

**Authors:** Mitchell C. Harris, Daniel C. DeRosa, Priscilla A. West

**Affiliations:** Department of Orthopaedic Surgery, Tripler Army Medical Center, Honolulu, HI, USA

## Abstract

Brodie's abscess is a subacute form of osteomyelitis which generally occurs in the metaphysis of the femur and tibia in the pediatric population. Pathogens are most commonly Gram-positive bacteria, notably Staphylococcus and Streptococcus. In this article, we describe a young pediatric patient presenting with subacute ankle pain with a subsequent diagnosis of Brodie's abscess of the talus secondary to Morganella morganii. We review the presentation, diagnosis, and treatment of this unique patient. To our knowledge, this is the first report of Morganella morganii as a cause of Brodie's abscess.

## 1. Introduction

We present a case report of Morganella morganii isolated from Brodie's abscess involving the talus in a 16-month-old, previously healthy female. Brodie's abscess is uncommon in the talus, especially in the pediatric population, with only a few patients reported in the literature [1–4]. This is the first reported case of Morganella morganii causing subacute osteomyelitis in a pediatric patient. Osteomyelitis secondary to Morganella is very rare but has been previously reported in adults, usually with a history of a medical comorbidity, trauma, or lengthy hospitalization [5–9]. Gram-positive bacteria are the most commonly isolated pathogens in cases of subacute osteomyelitis, but there are reports of increasingly more common Gram-negative bacterial infections, usually from Pseudomonas, Escherichia, Kingella, and Salmonella [10].

Brodie abscess was first described by Sir Benjamin Brodie in 1832 and is a localized form of subacute osteomyelitis that most commonly occurs in the metaphysis of long bones of the lower extremities in children and young adults [5, 7]. The condition is marked by an insidious onset with few symptoms, thus making the diagnosis difficult. Clinical presentation generally includes limping, intermittent pain with ambulation, and focal tenderness. Systemic symptoms and elevated inflammatory markers are often absent.

Radiographic findings of subacute osteomyelitis are seldom encountered in the early stages of infection, with a sensitivity of less than 20% [10]. Patients with subacute osteomyelitis will infrequently have radiographic findings within the first two weeks of infection [11, 12]. When abnormalities are found on radiograph, they are usually seen as enlarging and poorly defined lucent shadows with surrounding sclerosis [9]. Although a bone scan or CT scan can be used, MRI is the most sensitive form of advanced imaging, ranging from 80-100% [10]. On MRI, the central cavity has a low signal on T1-weighted images and a high signal on T2-weighted images [9]. There is often reactive hyperemia in the surrounding bone marrow and, less frequently, a “double-line sign” on T2-weighted images [9].

Initial diagnosis is made based on clinical suspicion and radiographic findings. Subacute osteomyelitis can be treated nonoperatively in some cases, but surgical management is frequently preferred by physicians in order to rule out a neoplastic process and to obtain a definitive diagnosis [6]. Additionally, surgical management allows obtainment of cultures and potential guidance of antibiotic treatment. Unfortunately, the responsible pathogen is only identified by culture in 29-61% of cases [6].

## 2. Case Report

CB is a 16-month-old, previously healthy, female that initially presented to her primary care physician two weeks prior to presenting to our orthopaedic clinic with limping and intermittent refusal to bear weight through the left leg. The mother of the patient denied any previous trauma or constitutional symptoms but did endorse foreign travel; they were living in Japan at the time of presentation to our department. The patient was current on all vaccinations.

The initial orthopaedic evaluation revealed a well-appearing, healthy child in no acute distress. The gait exam revealed that she refused to weight bear on the left lower extremity. The patient had very mild generalized tenderness in the left midfoot region; otherwise, no other area of tenderness was appreciated upon further examination of the lower extremities. She had full, painless range of motion of her hip, knee, and ankle joints. There was no erythema or swelling of the left foot; however, there was a mild effusion of the ankle. She was neurovascularly intact with normal reflexes.

**Figure 1:**
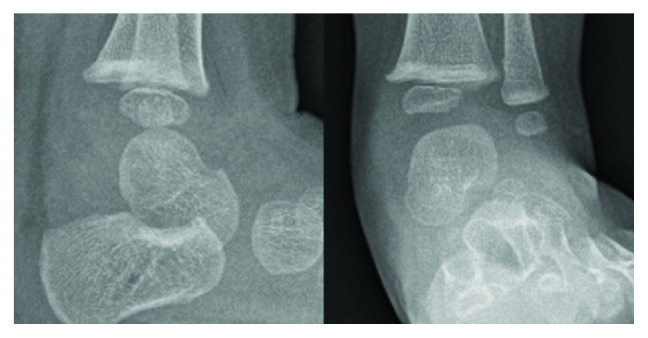
Lateral and AP plain radiograph of the ankle at presentation.

**Figure 2:**
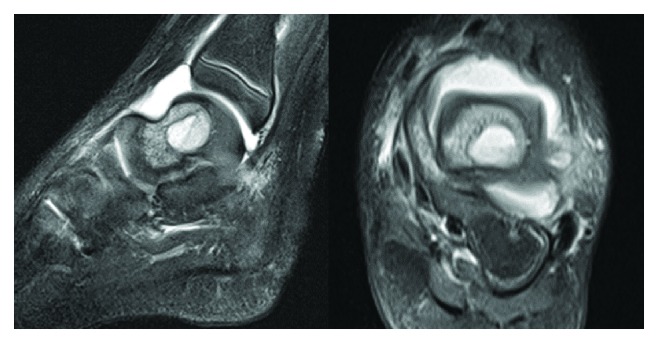
MRI T2-weighted lateral and axial cuts of the ankle.

**Figure 3:**
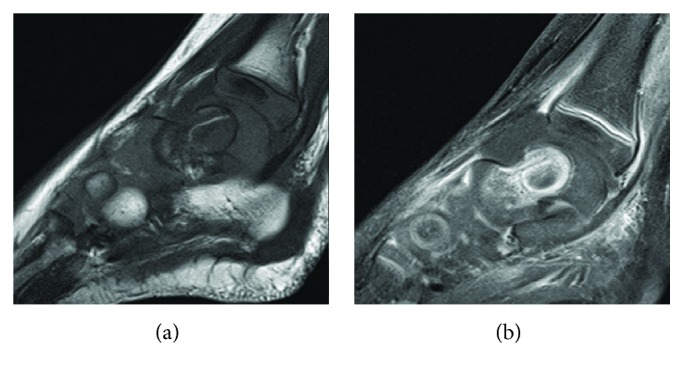
MRI T1-weighted lateral (a) and T1 postcontrast lateral (b) cuts of the ankle.

She was afebrile, and vital signs were within normal parameters. Radiographs of the left lower extremity revealed no osseous abnormality (Figure 1). Laboratory findings revealed a slightly elevated erythrocyte sedimentation rate of 34 mm/hr; otherwise, the white blood cell count (10,200 cells/*μ*L), differential (45% segmented neutrophils, no bands), and C-reactive protein (<0.05 mg/dL) were normal [8]. An MRI of her left ankle showed an ankle joint effusion, a 16 mm fluid collection with a high T2 signal with surrounding bone marrow edema, and a low signal on T1 (Figures 2 and 3). The findings were consistent with a Brodie abscess with surrounding osteomyelitis and a possible septic ankle. Furthermore, there was rim enhancement with gadolinium contrast, making an abscess more likely than a tumor (Figure 3) [13].

**Figure 4:**
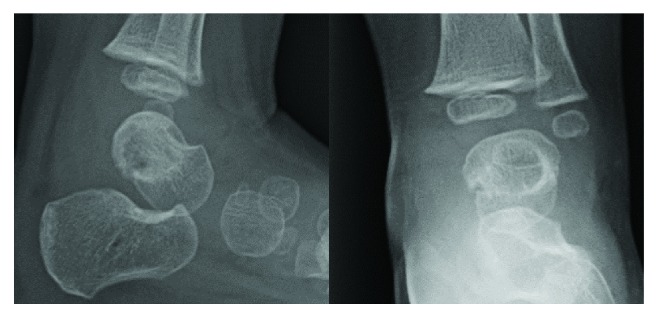
Lateral and AP plain films of the postoperative ankle.

The diagnosis and treatment were discussed with the parents, and she was consented for surgery. An anteromedial incision was used to approach the talus. Normal-appearing synovial fluid was encountered and cultured. The talus was then drilled through a nonarticular region with a 2.0 mm drill under fluoroscopic guidance, aiming in a posterolateral trajectory, resulting in an egress of purulent fluid which was sent for Gram stain and culture (Figure 4). The cavity was then thoroughly irrigated with saline, a Penrose drain was placed into the wound, and the incision was loosely closed around the drain. A long leg splint was applied to protect the soft tissues and prevent weight bearing. Infectious diseases was consulted, and the patient was admitted to the hospital for empiric intravenous antibiotics (Clindamycin) while awaiting aerobic/anaerobic, fungal, and acid fast bacilli cultures.

**Figure 5:**
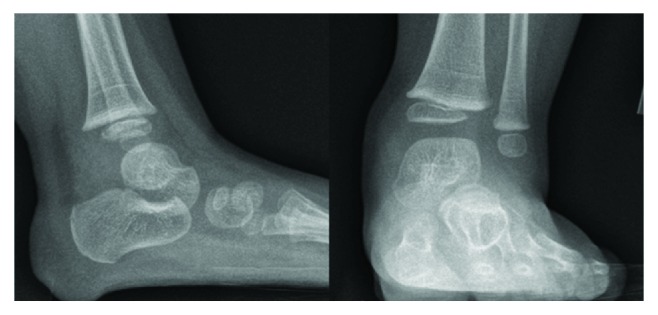
Lateral and AP plain films one year after I&D.

Gram stain of the purulent fluid revealed Gram-negative rods. Synovial fluid Gram stain and final cultures were negative, as were the fungal and acid fast bacilli cultures. On postoperative day (POD) #1, ceftriaxone was added to Clindamycin for Gram-negative coverage. On POD #2, aerobic and anaerobic cultures grew Morganella morganii; Clindamycin was discontinued, and the patient remained on ceftriaxone. Antibiotic sensitivities revealed resistance to ampicillin/sulbactam but sensitivity to cefepime, ciprofloxacin, gentamycin, and trimethoprim/sulfamethoxazole. On POD #4, she was transitioned to a three-week course of oral cefixime and discharged with close follow-up. She recovered well without any recurrence of symptoms at her scheduled postoperative visits (Figure 5).

## 3. Discussion

Brodie's abscess can be difficult to diagnose due to the insidious onset of symptoms, and diagnosis is frequently delayed by weeks to months. The differential diagnosis of a limping child is extensive, but with radiographic findings of an osseous lucency, the differential is narrowed to benign or malignant tumor versus infection. In our case, an MRI with contrast was useful since there were no radiographic findings on plain films, and inflammatory markers were equivocal.

The most common pathogens isolated after culture of Brodie's abscess include Staphylococcus, Streptococcus, and Kingella and less commonly Pseudomonas, Salmonella, Escherichia, and Haemophilus influenzae type b (Hib) [10]. Morganella morganii is a motile, facultative, anaerobic Gram-negative bacterium. An enteric bacterium, M. morganii, was first isolated from a pediatric fecal culture by Morgan and Liu et al. in 1906 [14, 15]. The bacterium is an opportunistic pathogen that rarely causes infection, except in the immunocompromised, but is notable for its increasing drug resistance in recent years [15]. Bacteremia, urinary tract infections, and abscesses are the most common pathologic manifestations of M. morganii infections, but osteomyelitis is exceptionally rare, and there are very few reports of such an infection [15–19].

Antistaphylococcal antibiotics are generally recommended for treatment of subacute osteomyelitis, but this has come into question with the increasing prevalence or detection of Gram-negative infections, such as K. kingae and Pseudomonas [20]. If Gram stain and cultures are obtained, as were done in our patient, the antibiotic can be quickly modified to cover the offending bacteria. However, some physicians argue for nonoperative treatment of subacute osteomyelitis and would need broader antibiotic coverage than is usually recommended or risk a poor clinical response in these patients [20, 21]. Nearly half of the patients also have negative cultures after surgical debridement, which may be indicative of a fastidious organism in some cases [6]. We believe this case adds to the available literature indicating that Gram-negative bacteria are becoming increasingly problematic as causative organisms in pediatric osteomyelitis [20, 22, 23]. Careful consideration should be given to using broader antibiotic coverage, particularly if being treated nonoperatively or if cultures are negative.

In our case, the culture revealed a Gram-negative and previously undescribed pathogen of Brodie's abscess in an uncommon location, i.e., the talus. The patient was successfully treated with surgical debridement, and a short stint of intravenous antibiotics followed by a three-week course of oral antibiotics. Gram-negative coverage was essential to treatment since this was not a typical case of subacute osteomyelitis. To our knowledge, this is the first report of Brodie's abscess due to Morganella morganii.
